# Supplementary material for: Mechanisms governing the pioneering and redistribution capabilities of the non-classical pioneer PU.1
Source: Nat Commun. 2020 Jan 21;11:402. doi: 10.1038/s41467-019-13960-2 (PMC6972792; doi:10.1038/s41467-019-13960-2)
Supplement: Supplementary file 7 — Source data [file 41467_2019_13960_MOESM7_ESM.zip › Source_Data/Figure5/Figure5A_MotifScanOutput/homerResults/motif19.similar.html]

motif19

## Information for motif19

G
A
C
T
A
G
C
T
C
G
A
T
A
C
T
G
G
T
C
A
C
A
G
T
A
T
G
C
C
G
A
T
C
G
A
T
G
C
T
A
C
G
A
T
T
G
A
C
  
Reverse Opposite:  

A
C
T
G
G
C
T
A
C
G
A
T
G
C
T
A
C
G
T
A
T
A
C
G
G
T
C
A
C
A
G
T
T
G
A
C
C
G
T
A
T
C
G
A
C
T
G
A
  

|  |  |
| --- | --- |
| p-value: | 1e-49 |
| log p-value: | -1.144e+02 |
| Information Content per bp: | 1.636 |
| Number of Target Sequences with motif | 346.0 |
| Percentage of Target Sequences with motif | 11.47% |
| Number of Background Sequences with motif | 2052.9 |
| Percentage of Background Sequences with motif | 4.50% |
| Average Position of motif in Targets | 224.8 +/- 144.4bp |
| Average Position of motif in Background | 202.7 +/- 128.9bp |
| Strand Bias (log2 ratio + to - strand density) | -0.3 |
| Multiplicity (# of sites on avg that occur together) | 1.10 |
| Motif File: | file (matrix) reverse opposite |

### Similar de novo motifs found

|  |  |  |  |  |  |  |  |
| --- | --- | --- | --- | --- | --- | --- | --- |
| Rank | Match Score | Redundant Motif | P-value | log P-value | % of Targets | % of Background | Motif file |
| 1 | 0.922 | G A C T G A C T C G A T A C T G G C T A C A G T A T G C G C A T G A C T C G T A C G A T G A T C G A C T | 1e-30 | -69.495981 | 5.74% | 1.98% | motif file (matrix) |
